# Lipid remodeling in response to methionine stress in MDA-MBA-468 triple-negative breast cancer cells

**DOI:** 10.1016/j.jlr.2021.100056

**Published:** 2021-02-26

**Authors:** Stacey L. Borrego, Johannes Fahrmann, Jue Hou, Da-Wei Lin, Bruce J. Tromberg, Oliver Fiehn, Peter Kaiser

**Affiliations:** 1 Department of Biological Chemistry, University of California, Irvine, Irvine, CA, USA; 2 West Coast Metabolomics Center, University of California, Davis, Davis, CA, USA; 3 Department of Clinical Cancer Prevention, University of Texas MD Anderson Cancer Center, Houston, TX, USA; 4 Department of Biomedical Engineering, University of California, Irvine, Irvine, CA, USA; 5 National Institute of Biomedical Imaging and Bioengineering, Bethesda, MD, USA

**Keywords:** cancer metabolism, homocysteine, methionine, methionine stress, lipid metabolism, lipid droplets, phospholipids, fatty acid metabolism, triglycerides, ASNS, asparagine synthetase, CARS, coherent anti-Stokes Raman scattering, CHOP, C/EBP Homologous Protein, DAG, diacyglycerol, Hcy, homocysteine, MB468, shortened for MDA-MB-468 triple negative breast cancer cells, MB468res-R8, Resistant cell line derived from MB468, also referred to as “R8”, Met, methionine, MS, methionine synthase, PC, phosphatidylcholine, PE, phosphatidylethanolamine, SAH, S-adenosylhomocysteine, SAM, S-adenosylmethionine, SRS, stimulated Raman scattering, UPR, unfolded protein response

## Abstract

Methionine (Met) is an essential amino acid and critical precursor to the cellular methyl donor S-adenosylmethionine. Unlike nontransformed cells, cancer cells have a unique metabolic requirement for Met and are unable to proliferate in growth media where Met is replaced with its metabolic precursor, homocysteine. This metabolic vulnerability is common among cancer cells regardless of tissue origin and is known as “methionine dependence”, “methionine stress sensitivity”, or the Hoffman effect. The response of lipids to Met stress, however, is not well-understood. Using mass spectroscopy, label-free vibrational microscopy, and next-generation sequencing, we characterize the response of lipids to Met stress in the triple-negative breast cancer cell line MDA-MB-468 and its Met stress insensitive derivative, MDA-MB-468res-R8. Lipidome analysis identified an immediate, global decrease in lipid abundances with the exception of triglycerides and an increase in lipid droplets in response to Met stress specifically in MDA-MB-468 cells. Furthermore, specific gene expression changes were observed as a secondary response to Met stress in MDA-MB-468, resulting in a downregulation of fatty acid metabolic genes and an upregulation of genes in the unfolded protein response pathway. We conclude that the extensive changes in lipid abundance during Met stress is a direct consequence of the modified metabolic profile previously described in Met stress–sensitive cells. The changes in lipid abundance likely results in changes in membrane composition inducing the unfolded protein response we observe.

Methionine (Met) metabolism is an integral aspect in cellular function and is particularly important in cancer. Cancer cells that cannot proliferate in growth medium when Met is replaced with its metabolic precursor, homocysteine (Hcy), have been characterized as being “methionine dependent” or “methionine sensitive” (1). Previous studies indicate that cancer cells that become resistant to Met stress or “methionine independent”, lose their transformed phenotype (2, 3, 4). Just as other metabolic alterations have been recognized as signatures of transformed cells, Met metabolism is a unique metabolic requirement of cancer and is referred to as the Hoffman effect (1, 5, 6).

Methionine is an essential metabolite and is the precursor to S-adenosylmethionine (SAM), the principal methyl donor in the cell. SAM serves as a co-factor for a variety of methyltransferases that catalyze methylation events on DNA, RNA, proteins, and lipids (7). After donating its methyl group, SAM is converted into S-adenosylhomocysteine and further processed to Hcy. At this point, Hcy can be catabolized in the transsulfuration pathway or methylated to regenerate Met by way of methionine synthase and 5-methyltetrahydrafolate or betaine homocysteine methyltransferase and betaine, a product of choline. Both SAM and betaine are direct components of Met metabolism and closely linked to phospholipid processing.

Phosphatidylcholine (PC) and phosphatidylethanolamine (PE) are the two most abundant lipid species in the cell and play an important structural role in cell membranes. Both PC and PE can be synthesized de novo through the two branches of the Kennedy pathway referred to as the CDP-choline and CDP-ethanolamine pathways, respectively (8, 9). In normal cells, the majority of PC is synthesized through the CDP-choline pathway. Choline links Met and PE metabolism as it is a precursor of betaine, which can regenerate Met by way of betaine homocysteine methyltransferase, and it can be regenerated by PC catabolism via phospholipase D (10). Interestingly, both choline and PC levels have been shown to be aberrantly upregulated in cancer along with many of their associated enzymes including phospholipase D (11, 12, 13, 14). In a less prominent pathway for PC synthesis, SAM is necessary for three sequential methylations on PE by the enzyme phosphatidylethanolamine N-methyltransferase (15, 16). In both human and yeast studies, atypical Hcy or S-adenosylhomocysteine levels lead to deregulation of phospholipid and triglyceride lipid metabolism (17, 18). The level in which these two pathways can influence each other indicate a tight balance between Met metabolism components and lipid synthesis.

The connection between Met and lipid metabolic pathways have been extensively researched in the context of liver dysfunction and heart disease in the presence of high Hcy levels. Recent work has extended these studies to different types of cancers of varying malignancy, and thus, the role of lipid metabolism and its connection to the Met pathway is becoming more clear in tumor progression and metastasis (13, 14, 19). In a continued effort to understand the metabolic response during Met stress, we performed lipidomic analyses in parallel to the untargeted metabolic and stable isotope-tracing study previously reported (4). Using the Met-dependent, triple negative breast cancer cell line MDA-MB-468 (MB468) and its Met-independent derived clone MDA-MB-468res-R8 (MB468res-R8 or R8), we have characterized the dynamic lipid response in cancer cells during Met stress.

## Materials and methods

### Cell lines, growth conditions, and treatments

MB468 and MB468res-R8 cell lines were maintained in DMEM (Sigma-Aldrich, D0422) supplemented with 10% dialyzed FBS (Omega Scientific), 1.5 μM cyanocobalamin (vitamin B12), 4 mM L-glutamine, 100 μM L-cysteine (Fisher Scientific), and 100 μM L-methionine (Sigma-Aldrich). In the case of methionine-free media, 370 μM DL-homocysteine (Sigma-Aldrich) was added in the absence of methionine.

To induce ER stress, MB468 and MB468res-R8 cells were treated with the 1 μM thapsigargin (Sigma-Aldrich, T9033) for 4 h. To inhibit PERK activation, cells were treated with 1 μM GSK2656157 (Millipore Sigma, 5.04651.0001) 1 h before media switch or thapsigargin treatment and replaced in the new media.

### Lipidome analysis

Lipidome analysis was performed by collecting 5 × 10^6^ cells per sample—pellet weights were measured for additional normalization. Each time point includes four biological replicates. Cell lysates were extracted as previously described (20). Briefly, 225 μl of chilled methanol containing an internal standard mixture [PE (17:0/17:0); PG (17:0/17:0); PC (17:0/0:0); C17 Spingosine; C17 Ceramide; SM (d18:0/17:0); Palmitic Acid-d3; PC (12:0/13:0); Cholesterol-d7; TG (17:0/17:1/17:0)-d5; DG (12:0/12:0/0:0); DG (18:half:0/0:0); MG (17:0/0:0/0:0); PE (17:1/0:0); LPC (17:0); LPE (17:1)] and 750 μl of chilled methyl tertiary butyl ether (Sigma-Aldrich) containing the internal standard 22:1 cholesteryl ester was added to cell lysates. Isotopically labeled internal standards included deuterated (d)-palmitate-d3, cholesterol-d7, and TG(17:0/17:0/17:0)-d5. Remaining lipid standards were selected as these were not identified in human plasmas during method development. We note that internal standards were used for normalization purposes and to correct for retention time drift, thereby increasing accuracy of annotations. Samples were shaken for 6 min at 4°C using an Orbital Mixing Chilling/Heating Plate (Torrey Pines Scientific Instruments) followed by the addition of 188 μl of room temperature distilled water. Samples were vortexed, centrifuged, the upper layer transferred to a new 1.5 ml microcentrifuge tube, and subsequently dried to completeness under reduced pressure. Upon complete dryness, samples were resuspended in methanol:toluene (90:10) with 50 ng/ml 12-[[(cyclohexylamino)carbonyl]amino]-dodecanoic acid (Cayman Chemical).

Lipid extracts were subsequently analyzed on an Agilent 1290A Infinity Ultra High Performance Liquid Chromatography system with an Agilent Accurate Mass-6530-QTOF in both positive and negative mode. The column (65°C) was a Waters Acquity UPLC CSH C18 (100 mm length × 2.1 mm internal diameter; 1.7 μM particles) containing a Waters Acquity VanGuard CSH C18 1.7 μM pre-column. The solvent system included A) 60:40 v/v acetonitrile:water (LCMS grade) containing 10 mM ammonium formate and 0.1% formic acid and B) 90:10 v/v isopropanol:acetonitrile containing 10 mM ammonium formate and 0.1% formic acid. The gradient started from 0 min 15% (B), 0–2 min 30% (B), 2–2.5 min 48% (B), 2.5–11 min 82% (B), 11–11.5 min 99% (B), 11.5–12 min 99% (B), 12–12.1 min 15% (B), and 12.1–15 min 15% (B). The flow rate was 0.6 ml/min and with an injection volume of 1 μl for ESI (+) and 5 μl for ESI (−) mode acquisition. ESI capillary voltage was +3.5 kV and −3.5 kV with collision energies of 25 eV and 40 eV for MS/MS collection in positive and negative acquisition modes, respectively. Data were collected at a mass range of m/z 60–1,700 Da with a spectral acquisition speed of 2 spectra per second. Method blanks and pooled bioreclamation plasma samples were included to serve as additional quality controls.

Data were processed using MZmine 2.10. All peak intensities are representative of peak heights. Peaks were annotated by matching experimental accurate mass MS/MS spectra to MS/MS libraries including Metlin-MSMS, NIST12, and LipidBlast (2). Spectral matching was automated using the MSPepSearch tool and manually curated using the NIST Mass Spectral Search Program v.2.0g. Metabolite libraries were created, in positive and negative ionization modes, containing all confirmed identified compounds. MZmine's Custom database search tool was used to assign annotations based on accurate mass and retention time matching using a m/z tolerance of 10 ppm and a RT tolerance of 0.1 min.

### Coherent anti-Stokes Raman scattering/stimulated Raman scattering microscopy

MB468 and MB468res-R8 cells were seeded at 200,000 cells per plate, 24 h before the start of the experiment in 35 mm glass bottom dishes (Fisher Scientific, NC9268399). Cells were rinsed twice with PBS before a media switch to methionine media (Met+) or homocysteine media (Met-Hcy+) for 0, 0.5, 2, 4, or 12 h. Cells were fixed before imaging by washing twice with PBS, treating with a 4% formaldehyde/PBS solution for 20 min at room temperature, washing with PBS three times, and storing cells at 4°C in PBS.

For lipid labeling experiments, MB468 and MB468res-R8 cells were seeded in 35 mm glass bottom dishes at 350,000 cells per plate, 12 h before the start of the experimental time course. Cells were cultured in either *unlabeled glucose media* or *labeled glucose media* prepared with 100 μM L-methionine or 370 μM DL-homocysteine. *Unlabeled glucose media* contains 4.5 g/L of glucose, and *labeled glucose media* contains 4.5 g/L of D-glucose-1,2,3,4,5,6,7-d_7_ (Sigma-Aldrich, 552003). Recipe for *base media*: DMEM without glucose (Caisson Labs, DMP04-10LT) supplemented with 10% dialyzed FBS (Omega Scientific), 1.5 μM vitamin B12, 4 mM L- glutamine, 1 mM sodium pyruvate, 44 mM sodium bicarbonate, and *50x amino acid mix.* Recipe for *50x amino acid mix*: L-arginine HCl 0.084 g/L, cysteine HCl 0.018 g/L, glycine 0.03 g/L, L-histidine HCl 0.042 g/L, L-isoleucine 0.105 g/L, L-leucine 0.105 g/L, L-phenylalanine 0.066 g/L, L-serine 0.066 g/L, L-threonine 0.095 g/L, L-tryptophan 0.016 g/L, and L-valine g/L.

Lipid synthesis analysis was performed by culturing cells in *unlabeled glucose media* with Met for 12 h, washing cells twice with PBS, adding *labeled glucose media* with Hcy for 0, 0.5, 2, 4, or 12 h, and then fixing with formaldehyde as described above. Lipid degradation analysis was performed by culturing cells in *labeled glucose media* with Met for 12 h, washing cells twice with PBS, adding *unlabeled glucose media* with Hcy, and then fixing with formaldehyde as described above.

Cells were imaged using a 76-MHz mode-locked Nd:vanadate laser that provides a beam at 1,064 nm functioning as the Stokes beam and a second harmonic generated beam at 532 nm to pump an optical parametric oscillator. The pump beam generated by the optical parametric oscillator is spatially and temporally overlapped with the Stokes beam and sent to the microscope. The two beams are focused on the cells through a 60×, 1.2 numerical aperture water objective lens. The generated coherent anti-Stokes Raman scattering (CARS) signals are collected through the condenser and focused onto a Hamamatsu photomultiplier tube with a 650 ± 50 nm bandpass filter in front. The stimulated Raman scattering (SRS) signals are collected and detected with a Thorlab photodiode (FDS1010, Thorlabs, Inc.) and a high O.D. bandpass filter (Semrock, Inc.) Both signals were measured to identify the cells and their morphology using the nonresonant background (CARS) and quantify lipid content (SRS, which is background free).

### Gene expression analysis

Total RNA from MB468 and MB468res-R8 cells was extracted using the RNeasy Plus Mini Kit (Qiagen, 74134), and library preparation was performed using the TruSeq RNA Library Preparation Kit v2 (Illumina, RS-122-2001 and RS-122-2002) with the ERCC RNA Spike-In mix (ThermoFisher, 4456740) to control for sample preparation variation. PolyA selected libraries were sequenced using single-end, 100 bp reads at the University of California, Irvine Genomics High Throughput Facility on an Illumina HiSeq 4000 system.

Raw reads were aligned to a custom reference sequence, defined as the union of the human reference genome (GRCh38/hg38, UCSC Genome Browser) and the ERCC spike-in sequences (http://tools.invitrogen.com/downloads/ERCC92.fa) using HISAT2 alignment software (21). Number of reads mapped to each gene feature was quantified by featureCounts in the Rsubread package, and unwanted sample variation was determined by RUVSeq (22, 23). Read counts were normalized, and differential gene expression analysis was performed using DESeq2 (24). Pathway enrichment analysis was conducted via gene-set enrichment analysis (25). Gene-set enrichment analysis was performed on the DESeq2 normalized expression signals via a running-sum statistic procedure to determine the enrichment of biological pathways from the Molecular Signature Database Hallmark Gene Set Collection (26).

### Real-time quantitative PCR analysis

Total RNA from MB468 and MB468res-R8 cells was extracted using the RNeasy Plus Mini Kit (Qiagen, 74134). Equal amounts (1.5 μg) of RNA was reverse transcribed using SuperScript II Reverse Transcriptase (Invitrogen) per manufacturer's instructions—with the exception of using 0.3 uL of SuperScript II per reaction. Gene expression was quantified using SYBR Green (Bio-Rad, 1725124) and Bio-Rad CFX Connect real-time PCR detection system. Primer sequences are as follows: CHOP (forward) 5′-AGAACCAGGAAACGGAAACAGA-3′, (reverse) 5′-TCTCCTTCATGCGCTGCTTT-3′; ASNS (forward) 5′-TGCACCATGTGTGGCATTTG-3′; (reverse) 5′-AGCAGCAGTTGGTGTATCCAT-3′. Relative gene expression to Met+ control sample was determined using CFX Maestro software (Bio-Rad).

## Results

### Methionine stress induces a global lipid response

Unlike normal, nontransformed cells, the majority of cancer cells are unable to survive in growth media where Met has been replaced with its metabolic precursor, Hcy (4, 27, 28, 29, 30). To elucidate the role of Met in cancer, we use a Met-dependent and Met-independent cell pair: MB468 and MB468res-R8, respectively. While reversion of a transformed, Met-dependent cell to a nontransformed, Met-independent phenotype is a rare event, MB468 cells are one of the few cell lines capable of such a reversion. As previously reported by Hoffman *et al.* (3), we were able to generate Met-independent clones by culturing the parental cell line, MB468, in Hcy media (Met-Hcy+) for a prolonged period of time (4, 30, 31). The revertant clone, MB468res-R8, was selected as a control for these studies as it most closely resembles the parental cell in both morphology and proliferation rate while exhibiting a nontransformed, Met-independent phenotype (4, 30).

**Fig. 1:**
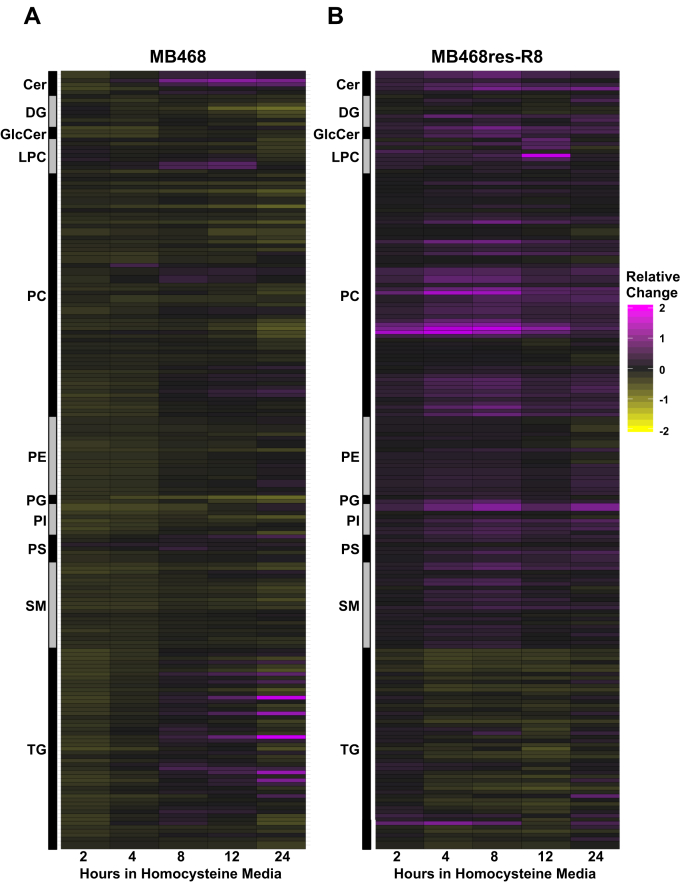
Homocysteine media induces a global lipid response in MB468 and MB468res-R8 cells. A: MB468 and (B) MB468res-R8 cells were cultured in Met+ or Met-Hcy+ media over the course of 24 h, measured by ultra-high performance liquid chromatography-quadrupole time-of-flight mass spectroscopy, and normalized to dry cell weight. Heatmaps indicating lipid classes are color filled based on relative change to the Met+, time-zero sample. Yellow indicates a decrease in metabolite abundance, and magenta indicates an increase relative to Met+, time-zero control. Cer, ceramide; DG, diglyceride; GlcCer, glucosylceramide; LPC, lysophosphatidylcholine; PC, phosphatidylcholine; PE, phophatidylethanolamine; PG, phosphatidylglycerol; PI, phosphatidylinositol; PS, phosphatidylserine; SM, sphingomyelin; TG, triglyceride.

Using MB468 and MB468res-R8 cells, we previously reported immediate metabolic changes in response to Hcy media in a cell line–specific manner (4). We further expanded on these metabolic findings and focused on the lipidomic response during Met stress. Both MB468 and MB468res-R8 cells were cultured in normal, Met media (Met+), or transitioned to Hcy media (Met-Hcy+) for 2, 4, 8, 12, and 24 h, and lipids were measured by ultra-high performance liquid chromatography-quadrupole time-of-flight mass spectroscopy (Fig. 1). We focus on early time points to avoid cell cycle effects imposed by the arrested growth of MB468 and continued proliferation of MB468res-R8 in Met-Hcy+ media (4, 30). Within 2 hours of exposure to Met-Hcy+ media, we observe a remarkable decrease in all lipid classes in MB468 cells (Fig. 1A). Interestingly, triglyceride abundance levels began a steady trend of recovery as early as 4 h postmedia switch, unlike the majority of lipid classes measured. In contrast to MB468 cells, the Met-independent MB468res-R8 cells show a global increase in lipid abundance with the exception of triglycerides (Fig. 1B). The decline in MB468res-R8 triglyceride levels is not as immediate as observed in MB468 cells, it takes up to 4 h in Met-Hcy+ media to observe a decline in the majority of triglyceride species. The contrast in global lipid behavior between the two cell lines may suggest differential coping mechanisms in response to Met stress or the remodeling of lipid behavior in MB468 as opposed to the expected general lipid increase/decrease trend for a proliferating cell in MB468res-R8. Importantly, these effects are initiated long before cell cycle arrest is induced and are thus autonomous responses in lipid metabolism characteristic for the Met-dependent state (30).

### Phosphatidylcholine and phosphatidylethanolamine abundance acutely decrease in response to methionine stress

**Fig. 2:**
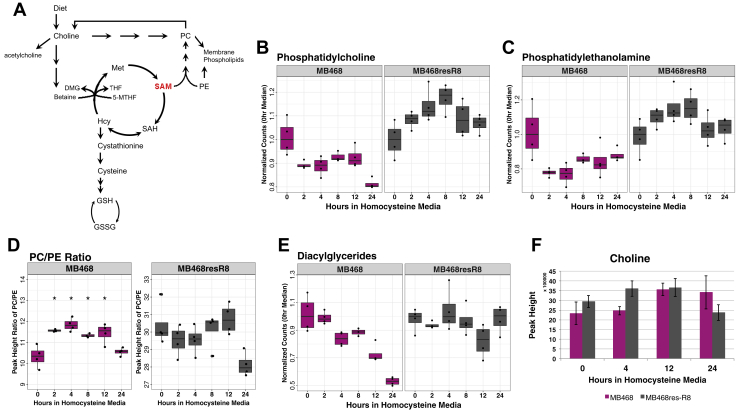
Phospholipids respond to homocysteine in MB468 cells. A: Schematic of homocysteine metabolism indicating the connection of SAM and choline in phospholipid synthesis. Three methylation reactions on PE using SAM as a co-factor are necessary to synthesize PC through the PEMT pathway. Choline is used as a precursor to PE in the Kennedy pathway. PC contributes to free choline through the enzyme phospholipase D. Figure adapted from Obeid et al. (32). Both MB468 (magenta) and MB468res-R8 (gray) were cultured in Met+ or Met-Hcy+ media over the course of 24 h, measured by ultra-high performance liquid chromatography-quadrupole time-of-flight mass spectroscopy, and normalized to dry cell weight. B: PC and (C) PE peak height values of all lipid species within each class were combined for each replicate and plotted for each time point. All time points are normalized to the median value of the Met+, time-zero sample. D: The ratio of PC/PE is plotted for MB468 and MB468res-R8 cell lines. Mean values of replicates for (A) PC and (B) PE were calculated for each time point, and PC mean values were divided by PE mean values for each time point. Statistical significance of difference between means was determined by Welch's *t*-tests and Benjamini-Hochberg procedure using the Met+, time-zero sample as a reference, *P*-values are indicated: ∗*P* ≤ 0.05. E: Diacylglyceride values were calculated and plotted as described above for PC and PE. F: Choline peak height values are shown at 0, 4, 12, and 24 h postmedia switch. DMG, dimethylglycine; GSH, reduced glutathione; GSSG, oxidized glutathione; Hcy, homocysteine; Met, methionine; 5-MTHF, 5-methyltetrahydrofolate; PC, phosphatidylcholine; PE, phosphatidylethanolamine; PEMT, phosphatidylethanolamine N-methyltransferase; SAH, S-adenohomocysteine; SAM, S-adenosylmethionine; THF, tetrahydrofolate.

Phospholipids are the largest class of lipids in the cell, and their synthesis is directly connected to Met metabolism (Fig. 2A). Both PC and PE are synthesized de novo in the endoplasmic reticulum via the Kennedy pathway using choline and ethanolamine, respectively (8). Choline not only serves as a precursor to PC but provides cellular betaine that can remethylate Hcy for Met regeneration (7, 33). As indicated by global lipid analysis (Fig. 1), MB468 and MB468res-R8 levels of PC (Fig. 2B) and PE (Fig. 2C) respond to Met-Hcy+ media in distinct fashions. In MB468 cells, PC and PE abundance levels drop below 90% and 80%, respectively, within 2 h postmedia switch. However, lipid abundances in MB468res-R8 cells indicate a steady increase over time and return to starting abundance levels by 24 h—following an expected lipid abundance trajectory for proliferating cells.

PE can be methylated three times by phosphatidylethanolamine N-methyltransferase using SAM as a methyl donor to synthesize PC (Fig. 2A) (9). Although newly synthesized SAM levels are reduced during Met stress, the PC/PE ratio increases within 2 h postmedia switch in MB468 cells (Fig. 2D) (4). This result excludes SAM limitation as a driving factor for PC and PE reduction, because if SAM were the limiting factor in PC synthesis, a decrease in the PC to PE ratio would be observed (34). However, the PC/PE ratio increase is likely because of a greater loss of PE levels than PC as previously noted. While declining abundance is observed in both PC and PE in response to Met-Hcy+ media, the considerable reduction of PE may indicate a greater sensitivity in this lipid class to Met stress as has been shown for ferroptosis (35).

It is interesting to note phospholipid abundances in time-zero samples cultured in Met+ media (Table 1). In breast cancer, PC and PE are detected at elevated levels as compared with normal cell and tissue samples, and fittingly, PE levels are approximately three times higher in MB468 than MB468res-R8 cells (36). In contrast, PC levels are comparable between the two cell lines. The increase in PE in MB468 may put these cells in a vulnerable state as the PC/PE ratio is modified and perhaps less resilient to stress, making the cells more susceptible to damage by oxidative stress and inducing an ER stress response by lipid disequilibrium (37).

**Table 1:** Mean peak height values of phospholipids in methionine media, time-zero samples

Lipid Species^a^	MB468 Mean	MB468 Std. Dev	MB468resR8 Mean	MB468resR8 Std. Dev	MB468/R8 Ratio^b^	*P*-Value^c^
PC	7.5e+07	5.5e+06	7.9e+07	6.1e+06	0.95	3.6e-01
PE	7.3e+06	9.1e+05	2.6e+06	2.7e+05	2.80	1.1e-03
PC/PE	1.0e+01	5.2e-01	3.0e+01	1.2	0.34	5.7e-06
PPC	2.5e+06	3.6e+05	1.2e+05	9.2e+03	20.44	8.6e-04
PPE	3.4e+06	6.1e+05	1.2e+06	1.1e+05	2.94	4.1e-03
PC/PPC	3.0e+01	1.9	6.3e+02	6.4	0.05	4.2e-08
PE/PPE	2.1	1.1e-01	2.2	4.5e-02	0.96	2.0e-01

PC, phosphatidylcholine; PE, phophatidylethanolamine.

a Lipid species were combined for each lipid class within each of the four replicates. These values were then used to calculate the mean values and standard deviations.

b The MB468 and MB468res-R8 ratios for each lipid class were calculated by taking the MB468 mean value and dividing by the MB468res-R8 (R8)∖mean value.

c Significance was calculated using a Welch's *t*-test (unpaired, two-sided, unequal variance, 95% confidence level) comparing the time-zero, control sample replicate values of MB468 and MB468res-R8.

Both diacyglycerol (DAG) and choline contribute to the biosynthesis of PC and PE in the Kennedy pathway in the ER (9). With its own unique metabolic response to Met-Hcy+ media, DAGs gradually decrease over time in MB468 cells, whereas little change is observed in MB468res-R8 cells (Fig. 2E). Interestingly, choline abundances increase after 12 h postmedia switch in MB468 and within 4 h in MB468res-R8—a similar response to Met-Hcy+ media as previously reported on reduced and oxidized glutathione (Fig. 2F) (4). The unique profile of DAGs in MB468 may reflect its depletion as the cell attempts to restore declining PC abundances and choline levels increase to meet the sudden demand of PC synthesis. Furthermore, DAG depletion could also be a result of its use as a precursor for other lipid species including phosphatidic acid and triglycerides (38).

### Oxidative stress–induced lipid peroxidation may influence lipid remodeling

The immediate decrease in global lipid abundances in response to Met-Hcy+ media likely indicates lipid damage. Lipid peroxidation is a process that targets unsaturated lipids in the presence of free radicals or prooxidants and is one potential route for lipid damage when cells are cultured in Met-Hcy+ media. In the presence of oxidative stress, prooxidants attack unsaturated lipids resulting in lipid radicals. Lipid peroxyl radicals are produced when oxygen reacts with the lipid radical, and these reactive lipid species propagate damage by reacting with more unsaturated lipids. Neither lipid radicals nor lipid peroxyl radicals are identified as the original lipid species because the mass (m/z) is altered, and therefore, a different spectrum for the newly formed oxidized lipid species is generated (39, 40). In our study, we did not employ mass spectrometry techniques to measure oxidized lipids; however, we have previously measured oxidized lipids in individual cells using fluorescence lifetime imaging and showed a rapid and dramatic increase in oxidized lipids when MB468 cells are shifted to Hcy medium (4). We therefore suggest that the immediate decrease in lipid abundance is because of an increase in oxidized lipid species that are no longer interpreted as the original lipid species. The lipid peroxidation reaction can be terminated in the presence of an antioxidant such as reduced glutathione, a cellular oxidant readily available to neutralize casualties of oxidation (41, 42).

As previously reported, both reduced and oxidized glutathione levels increase in response to Hcy media–induced oxidative stress in MB468 and MB468res-R8 cells (4). Interestingly, glutathione levels increase in MB468 cells by 12 h postmedia switch, whereas MB468res-R8 cells respond earlier within 4 h. This delayed glutathione response in MB468 may allow lipid peroxidation to occur, decreasing phospholipid abundances immediately. Upon glutathione upregulation in MB468, phospholipids attempt to recover to starting levels without success by 24 h postmedia switch (Fig. 2B, C). Upregulation of glutathione suggests a response to oxidative stress, which is further validated by oxidative stress assays in MB468 and MB468res-R8 cells that indicate a mild reduction in ROS levels over time in Hcy media (supplemental Fig. S1).

It is worth noting that MB468res-R8 cells may be able to further circumvent lipid damage and facilitate recovery with an increase in glutamine. As glutamine can contribute to the cysteine pool for GSH synthesis and thus cellular redox homeostasis, we observe an early increase in glutamine within 2 h postmedia switch (*P* = 0.007), which coordinates with our previous cysteine results (4). In MB468 cells, however, no significant changes are observed (ANOVA *P* = 0.747). It is likely that the increased abundance of glutamine also contributes to the spike at 2 h postmedia switch observed in alpha-ketoglutarate (*P* ≤ 0.001), citric acid (*P* = 0.01084), and malic acid (*P* ≤ 0.001) in MB468res-R8 cells. These concomitant increases in tricarboxylic acid cycle metabolites and precursors for lipid metabolism may help alleviate the consequences of Met stress in MB468res-R8 cells.

### Triglycerides have a distinct response to methionine stress

**Fig. 3:**
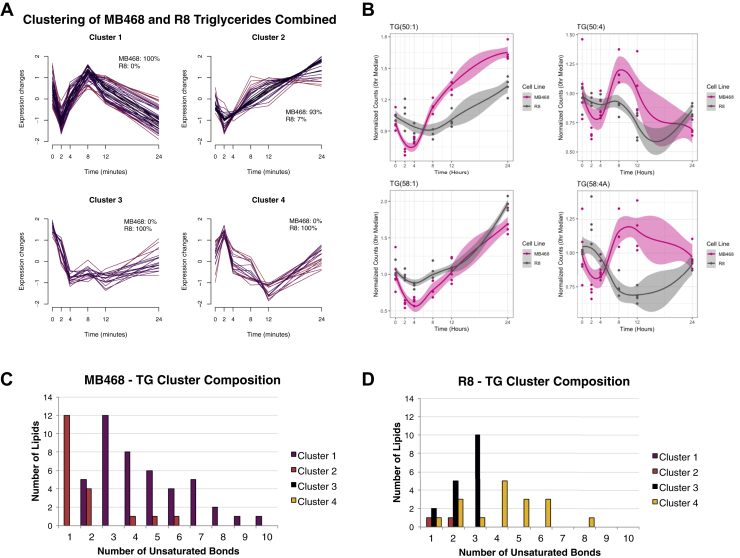
Triglycerides have a distinct response to methionine stress. A: Peak height data for all lipid species in the triglyceride (TG) class were combined for MB468 and MB468res-R8 (R8) cells. For each replicate, mean values were standardized, soft clustered using the R programming software MFuzz, filtered for alpha score values greater than 0.5, and plotted over time (43). The color gradient indicates the strength of the membership value for the cluster (black = strong, magenta = medium). The percentage of TGs from MB468 and MB468res-R8 (R8) in each cluster are indicated on each plot. B: Representative line plots of MB468 (magenta) and MB468res-R8 (gray) triglycerides with one or four unsaturated bonds and total tail lengths of 50 or 58 carbons. Confidence level of 0.75 is highlighted in corresponding line color. C: MB468 and (D) MB468res-R8 cells unsaturated TG lipids are plotted for each cluster in (A), regardless of total tail length.

An immediate global lipid response to Met stress is only observed in MB468 cells, yet both cell lines indicate a unique behavior in triglycerides as compared with other lipid species. Here, we focus on triglycerides from the lipidomic experiment summarized in Fig. 1. To understand the overall response of triglycerides over time in Met-Hcy+ media, all MB468 and MB468res-R8 triglyceride lipid abundances were clustered, and four distinct trends were identified (Fig. 3A) (43). Each cluster represents a unique trend, and there is remarkably minimal to no overlap of cell origin, confirming the distinct triglyceride response in Met-dependent and Met-independent cells. MB468 triglycerides typically decline in abundance within 2 h postmedia switch and attempt to recover by 8 (cluster 1) or 24 h (cluster 2) (Fig. 3A). MB468res-R8 cells also separate into two unique clusters, one with declining abundances by 4 h postmedia switch (cluster 3) and another that first increases in abundance at 2 h postmedia switch then decreases at 12 h (cluster 4) (Fig. 3A). These differences in triglyceride trajectories indicate that both cell lines have a unique response to Met-Hcy+ media and a unique feature of triglycerides segregates the cell-dependent response.

Triglycerides consist of a glycerol backbone and three fatty acid (FA) tails of varying chain length and unsaturated, double bonds. In order to determine any unique features of triglycerides that may be most affected during Met stress, correlation tests were performed on triglycerides in MB468 and MB468res-R8. We first tested lipid species with a combined tail length between 50 and 58 carbons and a single unsaturated bond. Our results indicate that both MB468 and MB468res-R8 triglycerides with a combined tail length of 50 carbons have unique profiles that only correlate with lipid species within each cell line (Pearson correlation coefficient r = 0.56) (Fig. 3B, top left panel). As the combined tail length increases to a combined length of 58 carbons, the profiles of both cell lines begin to look more similar (Pearson correlation coefficient r = 0.87) (Fig. 3B, bottom left panel). Next, we performed correlation tests on triglycerides in MB468 and MB468res-R8 with a combined tail length between 50 and 58 carbons and 4 unsaturated bonds. Our results indicate distinct profiles in MB468 and MB468res-R8 cells for lipid species with a combined tail length of 50 (Pearson correlation coefficient r = 0.03) (Fig. 3B, top right panel). Interestingly, at a combined tail length of 58 carbons, the lipid profiles in MB468 and MB468res-R8 become inversely correlated (Pearson correlation coefficient r = −0.68) (Fig. 3B, bottom right panel). These data suggest a particularly unique response of longer chain, heavily desaturated triglycerides in Met-dependent and Met-independent cells.

Further analysis of cluster composition indicates that the number of unsaturated bonds is the unique feature separating the triglyceride response to Met-Hcy+ media (Fig. 3A). Triglycerides in MB468 are primarily represented in clusters 1 and 2 (Fig. 3A); between these two clusters, cluster 1 mostly contains single unsaturated lipids, whereas cluster 2 contains lipid species with 3 or more unsaturated bonds (Fig. 3C). MB468res-R8 predominantly composes clusters 3 and 4 (Fig. 3A), with a distinct division of lipids in cluster 3 containing only lipid species with 1–3 unsaturated bonds and a range of unsaturated lipid species in cluster 4 (Fig. 3D).

These data indicate that MB468 and MB468res-R8 cells have unique lipid profiles in response to Met-Hcy+ over time regarding abundance levels. Both cell lines have unique profiles for lipid species with a single unsaturated double bond compared with four unsaturated, double bonds with little to no difference in profiles with the same number of unsaturated bonds but of varying combined tail lengths.

### Lipid droplets accumulate in MB468 in response to methionine stress

In addition to modified triglyceride behavior, an increase in lipid droplet abundance is also observed in MB468 cells in response to Met stress. Lipid droplets are dynamic organelles originating from the ER and comprised of a neutral lipid core of triglycerides and cholesterol esters surrounded by a phospholipid monolayer (44). Using CARS and SRS microscopy to specifically detect C-H bonds in FAs, we quantify lipid content per cell (45). Using this method, lipid abundances are measured as the number of pixels that belong to lipid droplets in a cell over the total pixel numbers of the entire cell (46).

**Fig. 4:**
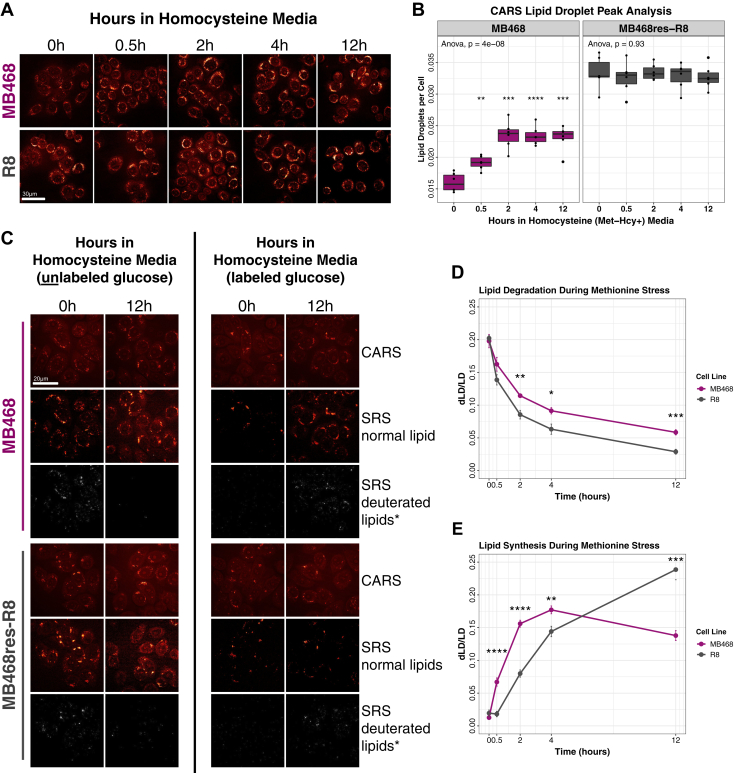
Lipid droplets respond to methionine stress in MB468 cells. A, Representative CARS images of lipid droplets in MB468 and MB468res-R8 cells cultured in Met+ (0 h) or Met-Hcy+ (0.5, 2, 4, and 12 h) media. The scale bar is 30 μm. B: MB468 (magenta) and MB468res-R8 (gray) samples were plated in duplicate, and lipid droplet values per cell from two fields of view per sample are plotted. One-way ANOVA results are reported at the top of each plot. Statistical significance of difference between means was determined by unpaired, two-tailed Student's *t*-tests and Benjamini-Hochberg procedure using the Met+, time-zero sample as a reference. Adjusted *P*-values are indicated: ∗∗*P* ≤ 0.01, ∗∗∗*P* ≤ 0.001, ∗∗∗∗*P* ≤ 0.0001. C: Representative CARS (total lipid droplets) and SRS (normal and deuterium-labeled lipids) images of MB468 and MB468-R8 cells at 0 h and 12 h postmedia switch. The scale bar is 20 μm. Left panel—lipid degradation assay: MB468 and MB468res-R8 cells were cultured in methionine (Met+) media supplemented with deuterium-labeled glucose for 12 h followed by a media switch to unlabeled Met+ (0 h) or unlabeled Met-Hcy+ (0.5, 2, 4, and 12 h) media. Right panel—lipid synthesis assay: MB468 and MB468res-R8 cells were cultured in unlabeled Met+ media (0 h) or labeled homocysteine (Met-Hcy+) media (0.5, 2, 4, and 12 h). Quantification of SRS deuterium-labeled lipid droplets normalized to SRS total lipid droplets in (C) are shown for (D) lipid degradation and (E) lipid synthesis assays. Statistical significance of difference between means was determined by unpaired, two-tailed Student's *t*-tests and Benjamini-Hochberg procedure using the Met+, time-zero sample as a reference. Adjusted *P*-values are indicated: ∗*P* ≤ 0.05, ∗∗*P* ≤ 0.01, ∗∗∗*P* ≤ 0.001, ∗∗∗∗*P* ≤ 0.0001. SRS deuterated lipid images in (C) marked with (∗) are adjusted with an 50% increase in contrast for visual purposes only. CARS, coherent anti-Stokes Raman scattering.

Both MB468 and MB468res-R8 cells were cultured in Met+ (0 h) or Met-Hcy+ media (0.5, 2, 4, 24 h), and fixed samples were analyzed by CARS microscopy (Fig. 4A, B). In normal culturing conditions (Met+), MB468res-R8 cells have twice the lipid content per cell of MB468 cells (*P* ≤ 0.001). However, lipid content per cell does not change in MB468res-R8 cells upon Met-Hcy+ media switch (ANOVA *P* = 0.93). In contrast, MB468 lipid content per cell increases within 30 min postmedia switch to Met-Hcy+, and elevated abundance levels remain stable after 2 h exposure to Met stress (ANOVA, *P* ≤ 0.001). It is important to note that both lipid droplets and triglycerides have been linked directly with oxidative phosphorylation, for which we observe a downregulation in both MB468 and MB468res-R8 cells (4, 47, 48). Thus, the differences observed in both triglyceride trends and lipid droplet accumulation in response to Hcy media are likely not a result of impaired oxidative phosphorylation alone.

To better understand the remodeling of lipid metabolism in MB468—specifically the acute decrease in lipid abundance (Fig. 1A) and increase in lipid droplets (Fig. 4A, B), we combined SRS and CARS microscopy methods with deuterium-labeled glucose as a lipid precursor to label and monitor lipid dynamics (49, 50). Two experiments were designed to understand the mechanism of lipid degradation and synthesis during Met stress. First, we measured lipid degradation in MB468 and MB468res-R8 by labeling lipids with deuterated glucose in Met+ media for 12 h then switching the cell cultures to unlabeled Met+ (0 h) or Met-Hcy+ media (0.5, 2, 4, 12 h) (Fig. 4C, left panel and Fig. 4D). Fixed samples were measured using SRS and CARS microscopy for deuterium-labeled lipid droplets and total lipid pool. Both cell lines were labeled to approximately ∼20% after the 12 h labeling period, and lipid content (deuterium-labeled lipid droplets/ total lipid pool) was normalized to the unlabeled, time-zero sample. Both cell lines presented similar decreasing trajectories over time, although MB468res-R8 cells lose their label faster within the first 30 min postmedia switch (MB468–0.16, MB468res-R8–0.13). However, labeled lipid content per cell ratios between the cells maintain a 0.03 difference over time. Thus, lipid degradation appears very similar in both Met-dependent and -independent cell lines.

Alternatively, lipid synthesis was determined by monitoring the increased presence of deuterium-labeled lipid content per cell over time. Both MB468 and MB468res-R8 cell lines were cultured in Met+ (0 h) or Met-Hcy+ media prepared with deuterium-labeled glucose (0.5, 2, 4, 12 h) and imaged by SRS and CARS microscopy (Fig. 4C, right panel and Fig. 4E). MB468 cells respond to the Met-Hcy+ media shift with a rapid increase in lipid synthesis up to 4 h postmedia switch as compared with MB468res-R8. However, lipid synthesis plateaus after 2 h in Met-Hcy+ media, whereas MB468res-R8 continues to synthesize lipids resulting in significantly more lipid synthesis after 12 h postmedia switch. With similar rates of lipid degradation between MB468 and MB468res-R8 cells, it appears that lipid synthesis is the predominate responding pathway to Met stress. The immediate acceleration of synthesis is likely a response to compensate for acute global loss of lipids (Fig. 1A), but the increased rate of synthesis is not sustained in Met-dependent cells, thus, leading to overall lower lipid production as compared with Met-independent MB468res-R8 cells (Fig. 4B).

### Gene expression during methionine stress

**Fig. 5:**
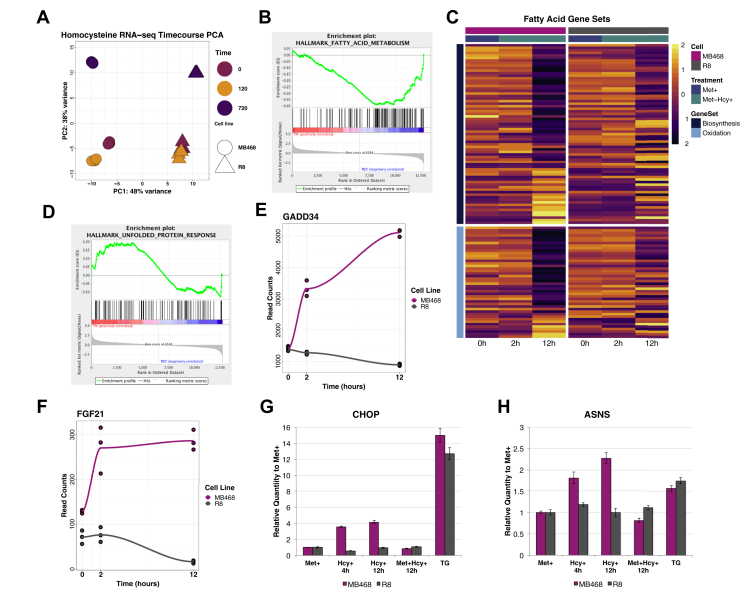
Gene expression during methionine stress. A: MB468 and MB468res-R8 cells were cultured in Met+ (0 h) or Met-Hcy+ (2 h and 12 h) media, and gene expression was analyzed by RNA-seq (single end, 100 bp read length, Illumina HiSeq 4000, n = 3). A principal component analysis plot indicates that MB468 (circle) and MB468res-R8 (triangle) cell lines have distinct genetic profiles but respond in a similar fashion to methionine stress. B: Gene set enrichment analysis (GSEA) of MB468 (red) and MB468res-R8 (blue) at 12 h postmedia switch indicates a negative correlation between the two cell lines in the fatty acid metabolism gene set. C: Heatmap representing GSEA gene sets *GO fatty acid biosynthetic process* (dark blue) and *GO fatty acid beta oxidation* (light blue). D: GSEA of MB468 (red) and MB468res-R8 (blue) at 12 h postmedia switch indicating a mixed response of genes in the unfolded protein response pathway. RNA-seq read counts for (E) GADD34 and (F) FGF21 in MB468 (magenta) and MB468res-R8 (gray), which are downstream targets of the UPR. Quantitative PCR results showing relative expression to Met+, control sample in MB468 (magenta) and MB468res-R8 (gray) for (G) C/EBP homologous protein (CHOP) and (H) asparagine synthetase (ASNS). Cells were cultured in Met+ (12 h), Met-Hcy+ (4, 12 h), Met+Hcy+ (12 h), or Met+/1uM Thapsigargan (TG, 4 h).

To further elucidate the metabolic response to Met stress, gene expression analysis was performed on MB468 and MB468res-R8 cells cultured in Met+ (0 h) or Met-Hcy+ media (2 or 12 h). Although MB468 and MB468res-R8 cells originate from the same genetic background, their expression profile in Met medium was surprisingly different. When these cells were shifted to Met-Hcy+ medium, expression programs in both cell lines were significantly remodeled to adapt to the modified growth conditions. Expression changes in response to Met stress were specific for MB468 and MB468res-R8 cells. Expression changes were only observed after prolonged culturing under Met stress, indicating that the observed rapid effects on lipid profiles were likely independent from transcription (Fig. 5A and Table 2). We applied gene set enrichment analysis using hallmark gene set collections and observed a prominent downregulation of FA metabolic genes in MB468 cells at 12 h postmedia switch as compared with MB468res-R8 cells (Fig. 5B) (25, 26, 51). Further analysis of gene ontology FA pathways indicated a 2-fold downregulation of 22.3% of genes in FA biosynthetic processes and 15.9% of genes in the FA beta-oxidation pathway at 12 h postmedia switch as compared with time-zero (Fig. 5C and Table 2). MB468 upregulated genes in FA biosynthetic and beta-oxidation processes are minimal—5.6% and 2.3%, respectively. As previously noted, effects on transcription are mostly delayed as exemplified by the gene expression profiles of FASN, ELOVL6, and ACLY in the FA biosynthetic processes pathway and CRAT, ACADM, and ACADS in the FA beta-oxidation pathway (Table 2). Therefore, it seems that a global shut down in lipid metabolism is a consequence of Met stress with a more pronounced effect in gene synthesis at 12 h postmedia switch.

**Table 2:** MB468 fatty acid metabolic genes upregulated or downregulated at 2 and 12 h postmedia switch as compared to time-zero

GO Pathway	Gene^a^	Fold Change—2 h	Fold Change—12 h
Biosynthetic process	PTGIS	0.88	0.19
Biosynthetic process	FADS1∗	0.97	0.22
Biosynthetic process	MSMO1	0.85	0.22
Biosynthetic process	EDN2∗	0.99	0.23
Biosynthetic process	FADS2∗	0.95	0.24
Biosynthetic process	PTGS1	0.76	0.24
Biosynthetic process	SCD	0.89	0.25
Biosynthetic process	AKR1C3∗	1.03	0.26
Biosynthetic process	FASN∗	0.98	0.30
Biosynthetic process	LTA4H∗	1.00	0.37
Biosynthetic process	ELOVL6∗	0.92	0.40
Biosynthetic process	FA2H∗	1.09	0.41
Biosynthetic process	GGT1	0.65	0.42
Biosynthetic process	ACLY∗	0.97	0.42
Biosynthetic process	ELOVL5∗	1.00	0.43
Biosynthetic process	ABCD3∗	0.93	0.45
Biosynthetic process	ALOX15∗	0.97	0.47
Biosynthetic process	OXSM∗	0.94	2.27
Biosynthetic process	MGLL∗	1.02	2.38
Biosynthetic process	MLYCD	0.89	2.40
Biosynthetic process	ALOXE3	2.12	11.43
Beta oxidation	CRAT∗	0.96	0.21
Beta oxidation	ACADS∗	0.90	0.37
Beta oxidation	ABCD3∗	0.93	0.45
Beta oxidation	ACADM∗	1.01	0.48
Beta oxidation	ECI2	0.89	0.48
Beta oxidation	BDH2∗	1.08	0.49
Beta oxidation	ACOX3	0.83	0.49
Beta oxidation	SESN2	4.86	8.10

a Genes marked with (∗) indicate fold change between 0.9 and 1.1 at 2 h postmedia switch.

The global lipid response, remodeled triglyceride behavior, decrease in lipid synthesis, and increase in lipid droplet abundance suggest that Met stress may largely affect functions of the ER. To further investigate this connection, we looked into the unfolded protein response (UPR), which is a cellular stress response pathway related to the ER and the ER stress response. Gene set enrichment analysis indicates no uniform response in UPR as subsets of genes are both upregulated and downregulated by 12 h postmedia switch (Fig. 5D). However, key genes found downstream of the UPR, including C/EBP homologous protein (CHOP), asparagine synthetase (ASNS), GADD34, and FGF21 were upregulated (Fig. 5E–H).

To understand the response of CHOP and ASNS, quantitative PCR was performed on MB468 and MB468res-R8 cells cultured in Met+, Met-Hcy+, or Met+Hcy+ medias. Cells were also treated with the ER stress inducing agent thapsigargin as a positive control (Fig. 5G, H). CHOP is a multifunctional transcription factor that responds to a wide variety of cellular stresses and is well-known to be upregulated during UPR and ER stress responses (52). As expected, CHOP was upregulated in response to thapsigargin in both MB468 and MB468res-R8 (Fig. 5G). Interestingly, CHOP was also upregulated in MB468, but not MB468res-R8, cells when cultured in Met-Hcy+ media. Furthermore, ASNS is transcriptionally regulated by activating transcript 4 and CHOP during the UPR and amino acid response (53, 54). While ASNS expression levels are moderately elevated in response to thapsigargin in both MB468 and MB468res-R8, a 2-fold upregulation is observed in MB468 cultured in Met-Hcy+ media (Fig. 5H). Both CHOP and ASNS upregulation is observed during Met stress in MB468 cells, suggesting full or partial activation of multiple stress response pathways, including ER stress, UPR, and the amino acid response.

## Discussion

We monitored lipid species during the initial phases of Met stress response in triple negative breast cancer cells MDA-MB-468. This cell line has been selected as a model because genetically largely identical, but Met-independent derivatives MB468res-R8 have been developed (4, 30, 31). We found extensive and fast lipid remodeling in response to Met stress in the breast cancer cell line MB468. We show an immediate, global decrease in lipid abundances with the exception of single unsaturated triglycerides, that was specific for Met-dependent cancer cells. Furthermore, lipid content per cell increases in response to Met stress specifically in MB468 but not MB468res-R8 cells. Gene expression changes are observed as a later response, indicating an overall decrease in FA metabolic genes and an upregulation of specific genes downstream of the unfolded protein response pathway, which was not observed in Met-independent cells.

Interestingly, numerous reports indicate that Hcy influences triglyceride biosynthesis and export, ER stress and UPR induction, and gene expression in cardiac and hepatic systems (55, 56, 57). Here, we see a comparable response profile in a cancer-specific setting that investigates the cell proliferation arrest and apoptosis when cancer cells are shifted to Met-Hcy+ medium. Notably, these effects of Hcy medium on cancer cells is not related to the presence of Hcy, but a manifestation of changed remodeled metabolism when Met is replaced by Hcy (6). This is also evident from UPR induction studies where addition of Hcy to MET containing medium has no effect on CHOP or ASNS induction, whereas Met-Hcy+ medium leads to CHOP and ASNS induction (Fig. 5G, H).

We have previously reported that Met stress in breast cancer cells can be rescued with SAM supplementation, suggesting that the impact on the one-carbon cycle and cellular methylation is causing a major cellular stress (30). We therefore expected to see an impact of PC levels because methylation of PE is hampered. However, we did not observe any change in the PC/PE ratio, but rather fast reduction of both phospholipid species specifically in MB468 cells. Our previous metabolite tracing studies suggested that cancer cells redirect Hcy to the transsulfuration pathway to increase the synthesis of glutathione and compensate for increased oxidative stress under these growth conditions (1, 4). We also observed an increase in lipid oxidation based on fluorescence lifetime imaging studies (4). We therefore hypothesize that the very rapid loss of several lipid species in MB468 cells may be because of a burst in lipid oxidation, which removes these species from detection by our mass spectrometric approach. We believe this is a likely scenario because lipid synthesis is rapidly increasing during this early phase of Met stress and is in fact more active in MB468 than in MB468res-R8 cells.

The robust effect on lipid content and the rapid change early after cancer cells were shifted to Hcy medium was surprising. Most of these effects are much earlier than gene expression effects are observed. It is therefore likely that the lipid composition in cancer cells during Met stress is a direct effect of the remodeled metabolism we observed in previous studies (4). Additional changes we observed in transcription profiles, lipid droplet accumulation, and UPR induction are likely adaptations or consequences of the initial impact by metabolic remodeling to adjust to Met stress. Future studies will be important to dissect signals that induce these adaptive changes to fully understand the role of lipid metabolism in the cancer-specific metabolic dependence on methionine.

## Data availability

All data are contained within the article—lipidomic results are attached as supplemental data. RNA-sequencing data and results have been deposited in the NCBI Gene Expression Omnibus (GEO) with accession number GSE155955.

## Supplemental data

This article contains supplemental data.

## Conflict of interest

The authors declare that they have no conflicts of interest with the contents of this article.
