# Supplementary material for: Lipid remodeling in response to methionine stress in MDA-MBA-468 triple-negative breast cancer cells
Source: J Lipid Res. 2021 Feb 26;62:100056. doi: 10.1016/j.jlr.2021.100056 (PMC8042402; doi:10.1016/j.jlr.2021.100056)
Supplement: Supplemental Fig. S1 [file mmc1.pdf]

SUPPLEMENTAL INFORMATION:

Lipid remodeling in response to methionine stress in MDA-MBA-468 triple-negative breast cancer cells

Stacey L. Borrego [1], Johannes Fahrmann [2, 3], Jue Hou [4], Da-Wei Lin [1],

Bruce J. Tromberg [4, 5], Oliver Fiehn [2], Peter Kaiser [1]

1 – Department of Biological Chemistry, University of California, Irvine, Irvine, CA

2 – West Coast Metabolomics Center, University of California, Davis, Davis, CA

3 – Department of Clinical Cancer Prevention, University of Texas MD Anderson Cancer Center, Houston, TX

4 – Department of Biomedical Engineering, University of California, Irvine, Irvine, CA

5 – National Institute of Biomedical Imaging and Bioengineering, Bethesda, MD

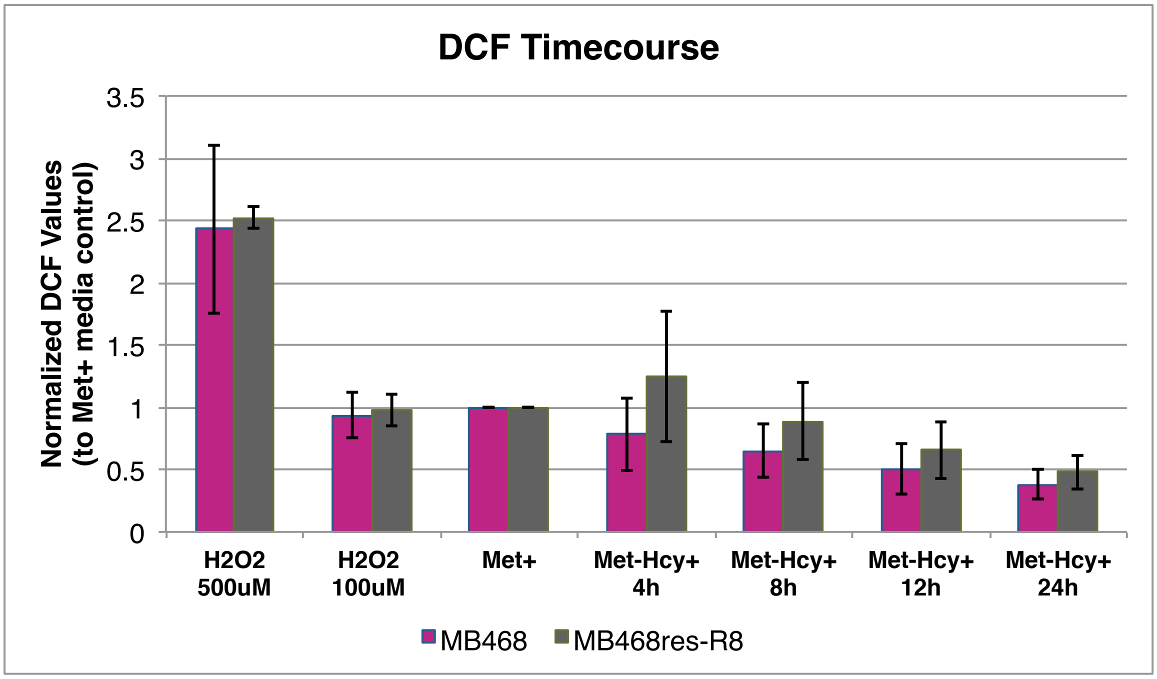

Supplemental Figure S1: Measure of reactive oxygen species in MB468 and MB468re-R8 cells

ROS levels were assessed by flow cytometry using the general oxidative stress indicator chloromethyl-H2DCFDA (Invitrogen, C6827) per manufacturer's instructions. Cells were cultured with hydrogen peroxide (H_2O_2) as positive controls, Met+ media as experimental control, and Met-Hcy+ media. The data represented are the medians of 3 experiments normalized to the median of Met+ control sample +/- standard deviation.
